# A comprehensive investigation of PRMT5 in the prognosis and ion channel features of lung cancer

**DOI:** 10.3389/fonc.2024.1478672

**Published:** 2024-11-29

**Authors:** Yan Wang, Daifang Chu, Haichao Li, Jiangjiang Fan, Ximing Zhu, Yulong Ma, Zhongping Gu, Nianlin Xie, Pengyu Jing

**Affiliations:** ^1^ Department of Thoracic Surgery, The Second Affiliated Hospital, Air Force Medical University, Xi’an, China; ^2^ Department of Pulmonary and Critical Care Medicine, The Second Affiliated Hospital, Air Force Medical University, Xi’an, China; ^3^ Department of Thoracic Surgery, Yicheng County People’s Hospital, Linfen, Shanxi, China

**Keywords:** lung cancer, PRMT5, prognosis, epigenetics, ion channel genes

## Abstract

The increasing incidence and mortality associated with lung cancer (LC) is a significant global health challenge. The underlying mechanisms contributing to LC remain inadequately understood. However, emerging evidence suggests that the epigenetic modifier protein arginine methyltransferase 5 (PRMT5) plays a complex role in various cellular processes, including DNA repair, gene transcription, and alternative splicing, through its function in catalyzing the symmetric dimethylation of both histone and non-histone proteins. In this study, we examined the functional role of PRMT5 utilizing LC-related datasets (GSE30219, GSE50081, and TCGA LC cohort) through a series of analyses. Our findings revealed that PRMT5 was significantly overexpressed in LC samples compared to normal tissues and was correlated with overall survival and disease-free survival rates. Additionally, PRDM1 was identified as a key protein exhibiting a strong interaction with PRMT5. The prognostic model that integrated PRMT5 with clinical factors demonstrated robust performance in assessing survival outcomes. Elevated levels of PRMT5 were associated with poor prognosis in LC, as evidenced by analyses of the GSE30219, GSE50081, and TCGA-LC datasets. Furthermore, we identified 27 ion channel (IC) genes exhibited a correlation with PRMT5 in lung adenocarcinoma (LUAD), of which 9 genes were identified as statistically significant with KM survival analysis. Strikingly, all of the 9 genes, including LRRC8A, the same as PRMT5, were associated with poor prognosis in LUAD. Our research highlights the potential of PRMT5 as a novel prognostic biomarker and its relationship with IC genes in LC.

## 1 Introduction

Lung cancer (LC) is a malignant tumor originating from the bronchial mucosa or gland of the lung tissues. It is widely recognized that lung cancer has emerged as the leading cause of cancer-related deaths (1). According to the clinical data published in the Lancet, the morbidity and mortality of LC have increased significantly and there are approximately 2 million new cases and 1.76 million death events reported annually (2), which poses the greatest threat to public health and well-being. Except for traditional treatment including surgery and chemotherapy, the advancement of therapeutic strategies aimed at genes exhibiting oncogenic alterations represents a significant milestone in the evolution of cancer treatment (3). Numerous targeted treatments have been developed great significance in clinics, including EGFR (4), CDK2 (5), ALK (6), RAD51 (7), IGFBPs (8), etc. Consequently, there is a strong impetus to establish a reliable biomarker that can effectively predict survival and therapeutic response in LC populations.

Arginine methylation represents a significant form of post-translational epigenetic modification (9). Protein arginine methyltransferase 5 (PRMT5) has been shown to exert a variety of functional effects on cellular processes, including the trajectories of cell differentiation and cell survival, by facilitating mono-methylation or symmetric dimethylation of both histone and non-histone substrates (10–12). The extensive role of PRMT5 is underscored by observations of embryonic lethality in mice lacking PRMT5 (13). As a key epigenetic regulator, PRMT5 is involved in numerous biological processes, such as DNA repair, alternative splicing, ribosome biogenesis, and cell proliferation (14).

A large number of data indicates a strong correlation between the activity of PRMT5 and the progression of aggressive tumors (15). PRMT5 has been reported to enhance cellular proliferation medicated by Wnt/β-catenin signaling through the epigenetic silencing of Dkk1 and DKK3, whereby PRMT5 facilitates the symmetric methylation of histones H3R8 and H4R3 in the promoter regions of Dkk1 and DKK3 (16). Consequently, PRMT5 has emerged as a critical therapeutic target in numerous malignancies, with small molecule inhibitors designed to reduce PRMT5 activity currently undergoing clinical trials, particularly in solid tumors and hematological cancers characterized by MTAP deletion (13, 17).

Research conducted over 30 years ago demonstrated that cancer cells exhibit a higher depolarized membrane potential in comparison to normal cells (18). Ion channel (IC) genes have been demonstrated to have significant impacts on the pathogenesis and progression of LC and their potential as targets for therapeutic intervention has also been assessed. Several drugs functioning as inhibitors of ICs were available, with some having undergone clinical trials for various types of cancers (19–22). Furthermore, due to their presence on cell membranes, IC genes serve as promising targets that can be utilized for diagnostic and therapeutic applications (23).

In this study, we examined the functional significance of PRMT5 in LC, investigated the upstream transcription factors involved, and characterized the downstream gene signatures and signaling pathways in LC cells with and without PRMT5 overexpression. Furthermore, we assessed the influence of PRMT5 on patient prognosis and established two distinct PRMT5 subgroups based on a diagnostic model derived from PRMT5 expression. Additionally, we analyzed the correlation between PRMT5 and IC genes, followed by an evaluation of the prognostic implications of PRMT5-related IC genes. Our findings underscore the critical role of PRMT5 in the prognosis of LC and its correlation with ICs.

## 2 Materials and methods

### 2.1 Data collection

LC-associated dataset GSE30219 was picked out from the Gene Expression Omnibus (GEO) database. We also acquired the clinical, RNA sequencing, and gene mutation data from TCGA-LC cohorts (mainly LUAD and LUSC) via GDC (https://portal.gdc.cancer.gov/). Samples lacking the complete clinicopathological data or gene expression were removed in our following analysis. A total of 293 LC samples and 14 normal samples were found in the GSE30219. Also, our original RNA-seq data which contained the transcriptome profiles between A549 cells overexpressing PRMT5 by transfecting a lentiviral vector (named PRMT5 OE group) and matching controls group (also termed as wild-type group, WT group) was also enrolled in our study. GSE30219 was used as a training dataset while GSE50081 and TCGA-LC cohort were used as two external validation datasets. Standardized counts were transformed into CPM data format by using the CPM function in the edgeR package.

### 2.2 Differentially expression analysis

The differential expression analysis was carried out by using the limma package. Then differentially expressed genes (DEGs) in different comparison cohorts (LC vs normal groups and PRMT5 OE vs WT group) were identified. The DEGs between LC and normal group followed with the threshold: the absolute value of fold change >1.5 and adjusted P value <0.05 while the DEGs between PRMT5 OE and WT group met the requirement: the absolute value of fold change >2 and adjusted P value <0.01. The heatmap and volcano plots were used to visualize the results. Notably, the expression data of PRMT5 was extracted from DEGs lists. Based on the median PRMT5 expression level, we categorized LC samples in GSE30219 into high PRMT5 and low PRMT5 groups. Likewise, the DEGs between high PRMT5 and low PRMT5 groups were also obtained for the following analysis.

### 2.3 Protein-protein interaction network analysis

To explore the upstream and downstream regulatory mechanism of PRMT5, TF targets of PRMT5 were predicted by ENCODE and hTFtarget online tools. 32 common TFs were identified by Venn analysis. STRING online tool was used to analyze the PPI of PRMT5 and the association of combined score > 0.7 was selected as the threshold of PPI to explore the candidate genes that had crosstalk with PRMT5. We take the intersection analysis between 32 common TFs and DEGs between PRMT5 OE and WT group to screen out the overlapping significant TFs and 3 differentially expressed TFs were detected. Likewise, Venn analysis between genes interacting with PRMT5 and DEGs between PRMT5 OE and WT groups was performed and 1 differentially expressed interaction gene PRDM1 was ascertained. Cytoscape was used to construct the 3 TFs-PRMT5 interaction network and PRMT5-protein interaction network.

### 2.4 Functional enrichment analysis

Both Gene Ontology (GO) and Kyoto Encyclopedia of Genes and Genomes (KEGG) pathway analyses were carried out to better clarify the characteristics of DEGs between PRMT5 OE and WT groups, which was helpful in digging out the predominant pathways and gene targets of PRMT5 in LC progression. Gene Set Enrichment Analysis (GSEA) is a computational method that is used to determine whether a given gene set has significant differences between different gene sets. Here, we investigated the potential signaling axis of DEGs between high PRMT5 and low PRMT5 groups by GSEA analysis.

### 2.5 Survival analysis

The impact of PRMT5 on LC survival was examined by using the survival package in R according to the survival information. Kaplan Meier (KM) curves were used to display the overall survival (OS) probability and disease-free progression (DFS) probability of each patient and the survival differences between high PRMT5 and low PRMT5 groups were compared by log-rank test. Univariate cox regression analysis was conducted to explore whether the expression level of PRMT5 could act as a prognostic risk factor. Receiver operating curve (ROC) analysis was carried out to explore the predictive accuracy of PRMT5 as a prognostic index. Moreover, we introduced the GSE50081 dataset and TCGA cohorts to check the predictive ability of PRMT5 in LC by conducting ROC analysis relying on the clinical survival data of LC patients in GSE50081 and TCGA-LC cohorts. Additionally, we also focused on the survival of ICs-related genes, which played a dispensable role in membrane potential effect and anti-tumor response.

### 2.6 Correlation analysis between clinical features and PRMT5 in LC

The differences in different clinical features such as TNM stage, pathological grades, gender, and ages between high PRMT5 and low PRMT5 groups were also surveyed based on the GSE30219 dataset. Wilcox test was used to detect the differences of continuous data and the Chi-square test was used to compare the non-continuous data. The percentages of each clinical parameter subpopulation between two PRMT5 groups were visualized in the box plot.

### 2.7 Nomogram analysis

Nomogram analysis formulates the scoring standard according to the regression coefficients of all independent variables such as age, gender, tumor size, TNM stages, clinical pathological grading, and so on (24). For each patient, a risk score can be calculated by the nomogram scoring system, which was then transformed into the probability of survival time of each patient. Nomogram is a powerful platform that shows great promise for personalized medicine and therapy (25). Here, we performed a nomograph analysis of PRMT5 by integrating other clinical factors that impact survival. Then ROC analysis was carried out according to the nomogram risk value. Notably, we divided LC samples into low and high-risk groups according to the median nomogram risk score. Survival analysis between two risk subgroups was conducted to examine the predictive power of the nomogram model. The calibration curve survey was used to validate the prognostic effect of the nomogram model.

### 2.8 Pearson correlation analysis

IC-related genes were screened and recorded through extensive literature review, and then compared with genes downloaded from the HUGO Gene Nomenclature Committee (HGNC) database. Subsequently, based on the database of UCSC XEAN (https://xena.ucsc.edu/), the pearson correlation was analyzed between IC-related genes and PRMT5 in TCGA-LUAD gene expression data. Screening genes with | r2 |>0.4 as IC genes related to PRMT5 expression.

### 2.9 RNA extraction, and quantitative real-time polymerase chain reaction

TRIzol reagent (Invitrogen) was used to extract RNA according to the manufacturer’s protocol. After isolation, 1 μm RNA was reverse-transcribed into complementary DNA (cDNA). To examine the expression of VDAC1/2/3 and CLIC1, qPCR was performed using Advanced SYBR Green supermix, with actin as the internal control. The primer sequences are as follows: VDAC1-F: ACCCACGTATGCCGATCTTG, VDAC1-R: AATGTGCTCCCGCTTGTACC. VDAC2-F: CGGGGTCCTGTCTGGGAGAG, VDAC2-R: GCTGGAGGGCGAAGTGAAGG, VDAC3-F: TGACTCTTGATACCATATTTGTACCG, VDAC3-R: GGTTGTACCACA ACGCACTAA. CLIC1-F: GTATTGACCAGTCTCCCTTCCAGC, CLIC1-R: GGTCTTTATGAGGAGGTCGTGGG.

## 3 Results

### 3.1 High expression and TF targets of PRMT5 in LC

**Figure 1:**
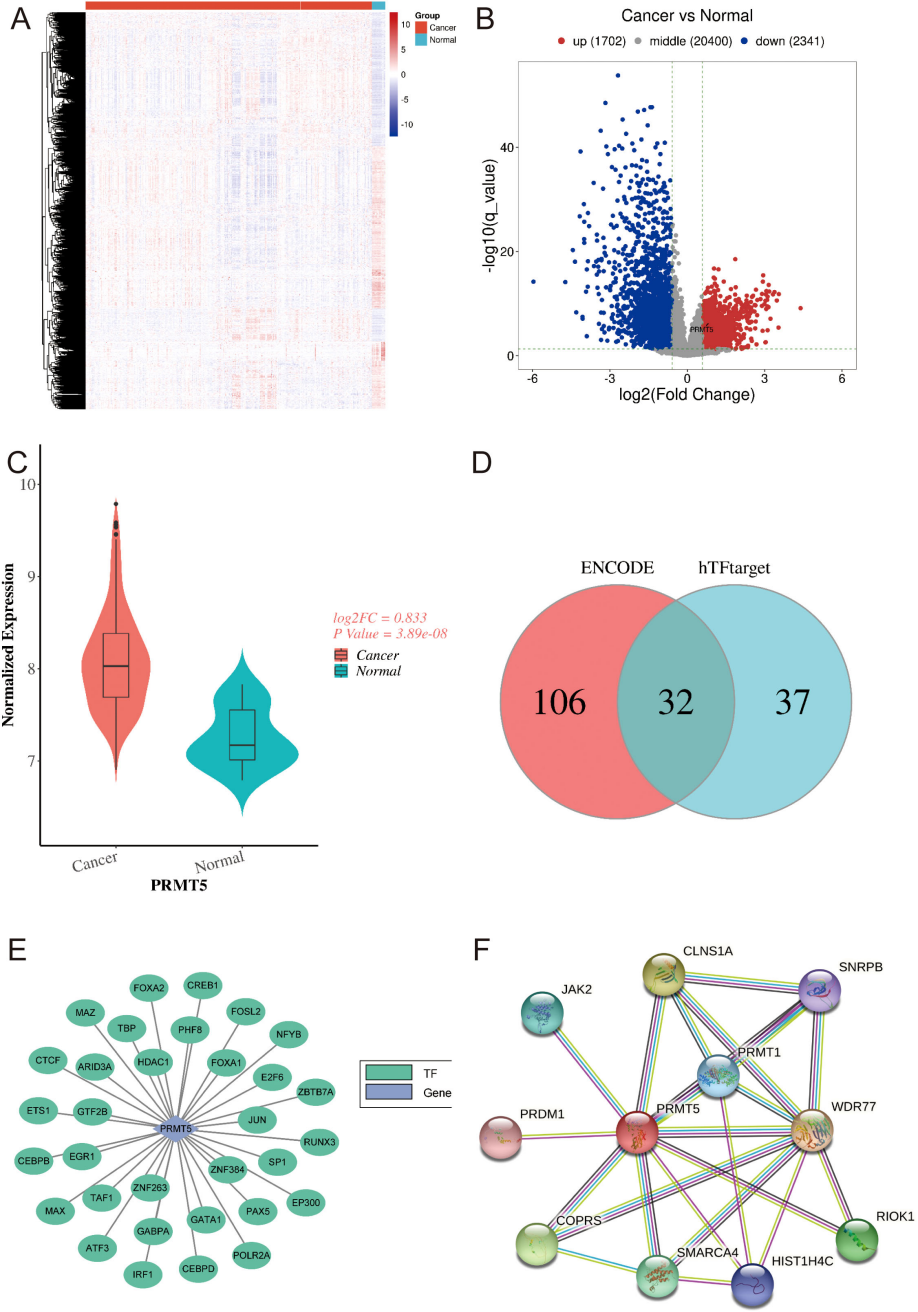
Expression features and TF targets of PRMT5 in LC. **(A)** The heatmap of 4043 DEGs in LC and normal group. **(B)** The volcano plot of 4043 DEGs in LC and normal group. **(C)** PRMT5 expression between LC and normal group. **(D, E)** Venn results of TFs targets of PRMT5 predicted by ENCODE and hTFtarget databases. **(F)** PPI network of PRMT5.

Here, 4043 DEGs were identified in GSE30219, which contained 293 LC samples and 14 normal samples (**Figures 1A, B**). 1702 DEGs were up-regulated while 2341 DEGs were down-regulated in LC. Notably, we observed that PRMT5 was increased in LC compared with the normal group (**Figure 1C**). Transcription factor (TF) has been verified to emerge as an effective governor in gene expression. Here, we found 32 overlapping TFs of PRMT5 by using the ENCODE and hTFtarget databases (**Figures 1D, E**). Furthermore, we also investigated the PPI network of PRMT5 using the STRING tool (**Figure 1E**). 10 genes were found to have interactions with PRMT5 (**Figure 1F**).

### 3.2 Function enrichment analysis of DEGs in LC cell overexpressing PRMT5

**Figure 2:**
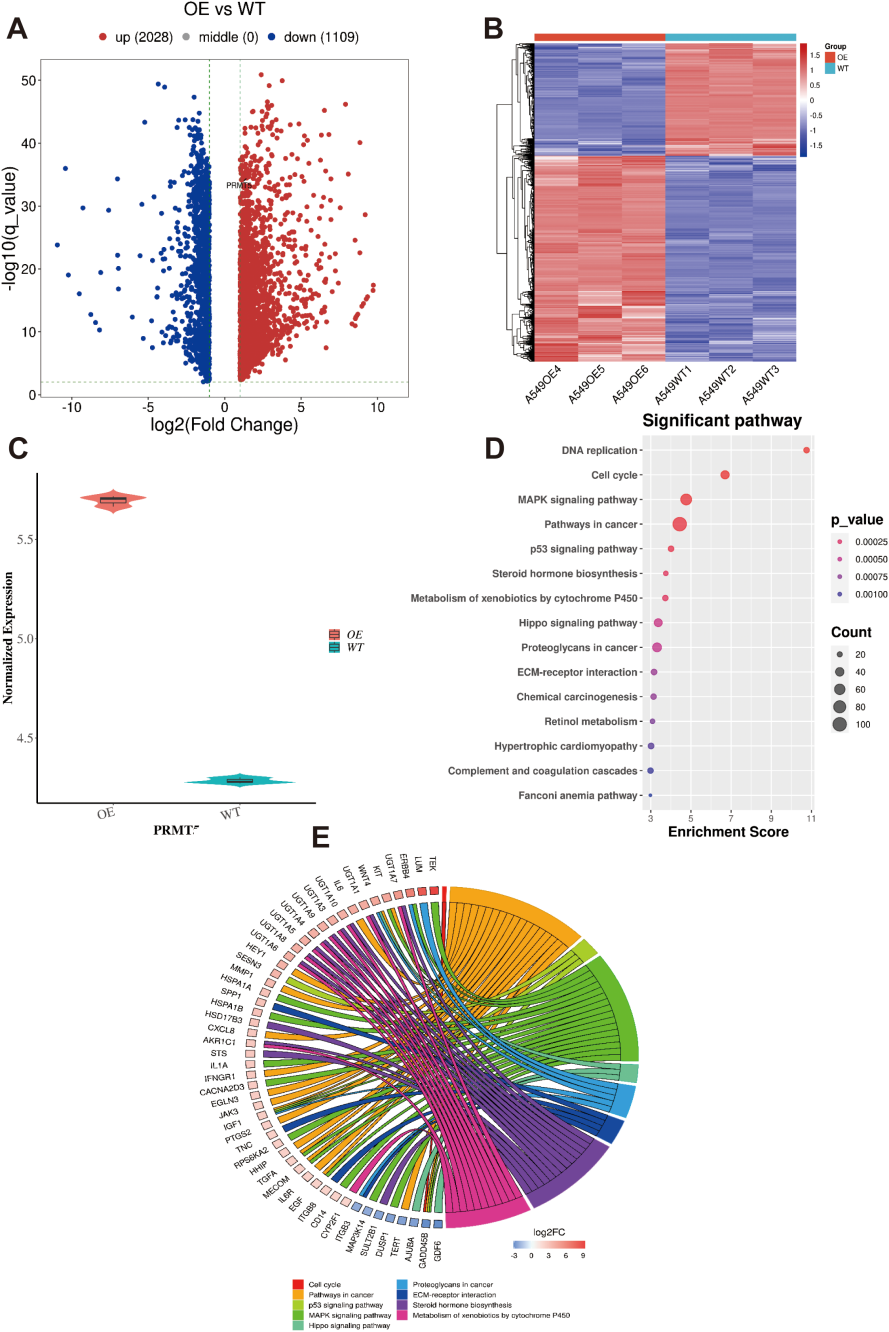
GO and KEGG analysis of DEGs in LC cell overexpressing PRMT5. **(A, B)** The heatmap and volcano plot of 3137 DEGs between OE and WT groups. **(C)** PRMT5 expression between OE and WT groups. **(D)**. GO analysis the of DEGs between PRMT5 OE group and WT. **(E)**. The circle plot of DEGs in terms of immune-related pathways.

Given the high expression of PRMT5 in LC, we generated A459 cells overexpressing PRMT5 (OE) and matching control group (also named wild-type, WT group). By analyzing the transcriptome data originating from OE and WT groups, 3137 DEGs were identified between OE and WT groups (**Figures 2A, B**). Among them, 2028 DEGs were upregulated while 1109 DEGs were down-regulated in LC. Of course, PRMT5 was enhanced in the OE group, which suggested that our cell line acquired the ability to overexpress PRMT5 lastly (**Figure 2C**). GO and KEGG enrichment analysis of the DEGs between the PRMT5 OE group and WT group was carried out to decipher the underlying mechanism of PRMT5 in influencing LC progression. Here we focused on the immune-related pathways because of the well-defined impact of immune response in cancer. DEGs were mainly associated with cancer associated with pathways, DNA repair, cell cycle, and MAPK signaling pathway. (**Figures 2D, E**).

### 3.3 Identification of 4 potential TF targets of PRMT5

**Figure 3:**
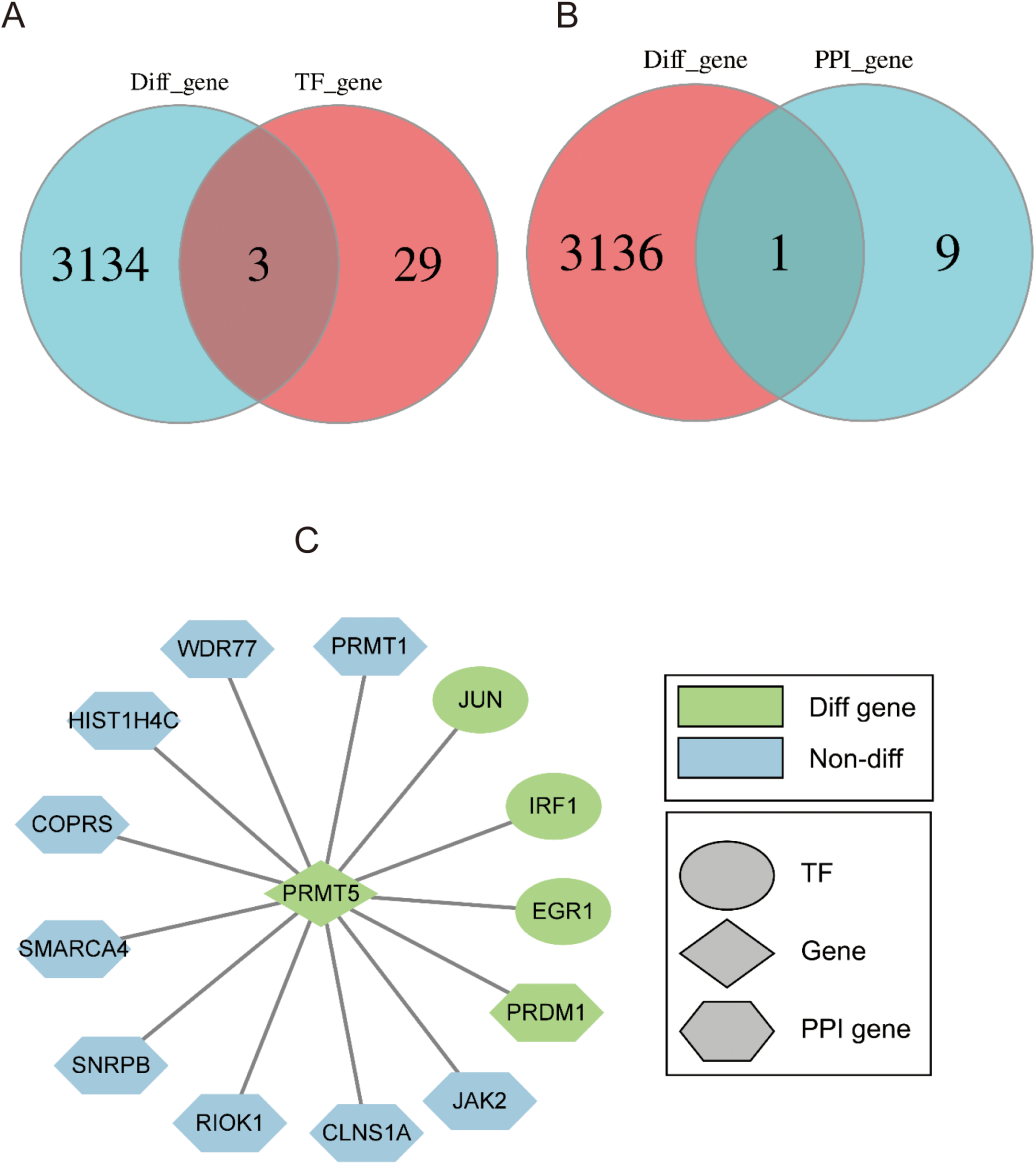
Identification of 4 potential TF targets of PRMT5. **(A)** Venn analysis of TFs and DEGs between PRMT5 OE and WT group. **(B)** Venn diagram of DEGs and 10 interaction genes. **(C)** The PPI network of PTMT5 involved key TFs and interaction genes.

To figure out the significant TFs from 3137 DEGs between PRMT5 OE and WT group, Venn analysis was performed. 3 shared TFs (EGR1, IRF1, and JUN) were identified (**Figure 3A**). EGR1, IRF1, and JUN could be potential driving factors to promote PRMT5 expression.1 overlapping gene (PRDM1) was identified between 10 PPI interaction genes with PRMT5 and 3137 DEGs (**Figure 3B**). There was a close relationship between PRMT5 and PRDM1. In summary, we constructed a PPI network of PTMT5 to get a landscape of upstream and downstream regulation profiles by integrating the differentially expressed TFs and interaction genes (**Figure 3C**).

### 3.4 PRMT5 was a negative regulator in LC prognosis

**Figure 4:**
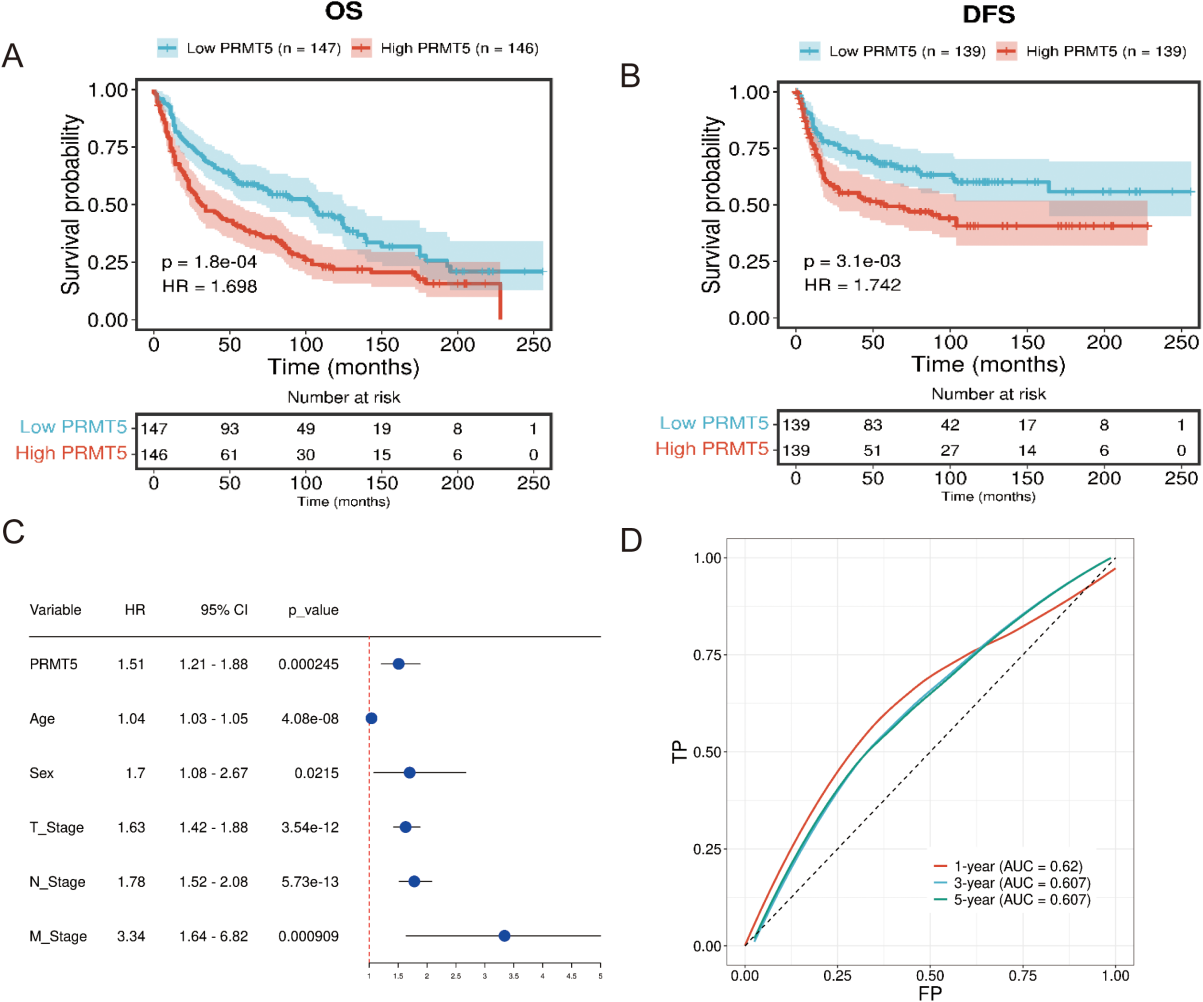
PRMT5 was a negative regulator in LC prognosis. **(A, B)** OS and DFS analysis of PRMT5 in GSE30219. **(C)** Univariate cox regression analysis of PRMT5 could act as an independent prognostic biomarker. **(D)** ROC of PRMT5 at predicting 1-, 3-, and 5-year overall survival.

PRMT5 was found to be associated with overall survival (OS) and disease-free survival (DFS) in LC according to the clinical data in GSE30219 (**Figures 4A, B**). PRMT5 had a significant effect on the prognosis of LC and patients with a high PRMT5 expression had poor survival. Univariate cox regression analysis indicated that PRMT5 could act as an independent prognostic biomarker (**Figure 4C**). These results revealed that PRMT5 was an unfavorable factor in LC prognosis. Furthermore, we examined the predictive power of PRMT5 in survival evaluation. ROC analysis was carried out to check the predictive accuracy of PRMT5 as a prognostic marker. The results showed that PRMT5 did the best predictive performance at predicting 1-, 3-, and 5-year overall survival (**Figure 4D**).

### 3.5 Verification of excellent predictive performance of PRMT5

**Figure 5:**
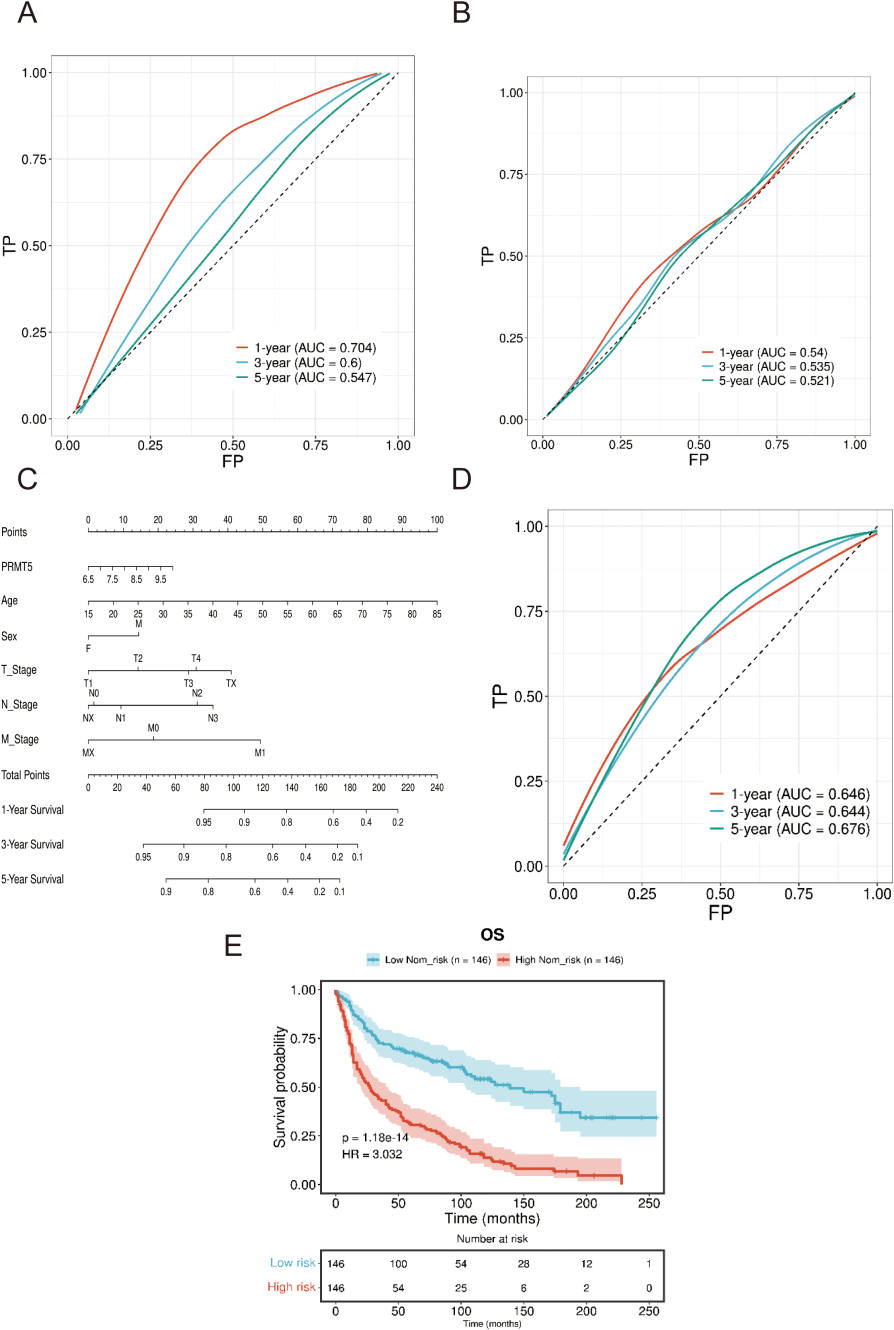
Verification of excellent predictive performance PRMT5. **(A, B)** ROC analysis of PRMT5 at predicting 1-, 3-, and 5-year survival in both GSE50081 and TCGA data. **(C)** Nomogram analysis of PRTM5 by univariate cox regression. **(D)** ROC analysis of PRMT5 at predicting 1-, 3-, and 5-year survival by nomogram. **(E)** Survival analysis between high and low-risk groups.

Furthermore, we examined the predictive power of PRMT5 in GSE50081 and TCGA (TCGA-LUAD and TCGA-LUSC) clinical data. ROC analysis manifested that PRMT5 displayed excellent predictive ability in predicting 1-, 3-, and 5-year survival in both GSE50081 and TCGA data (**Figures 5A, B**). Here, we introduced nomogram analysis to check the prognostic significance of PRTM5 by univariate cox regression. Results confirmed the independent factor potential of PRMT5 (**Figures 5C, D**). Accordingly, we divided LC samples in GSE50081 data into high and low-risk groups based on the median risk score identified by the nomogram. Survival analysis indicated that the high-risk group had worse survival time compared with the low-risk group (**Figure 5E**), which suggested the predominant influence of PRMT5 in prognosis once again.

### 3.6 Differences of clinical features in high and low PRMT5 groups

**Figure 6:**
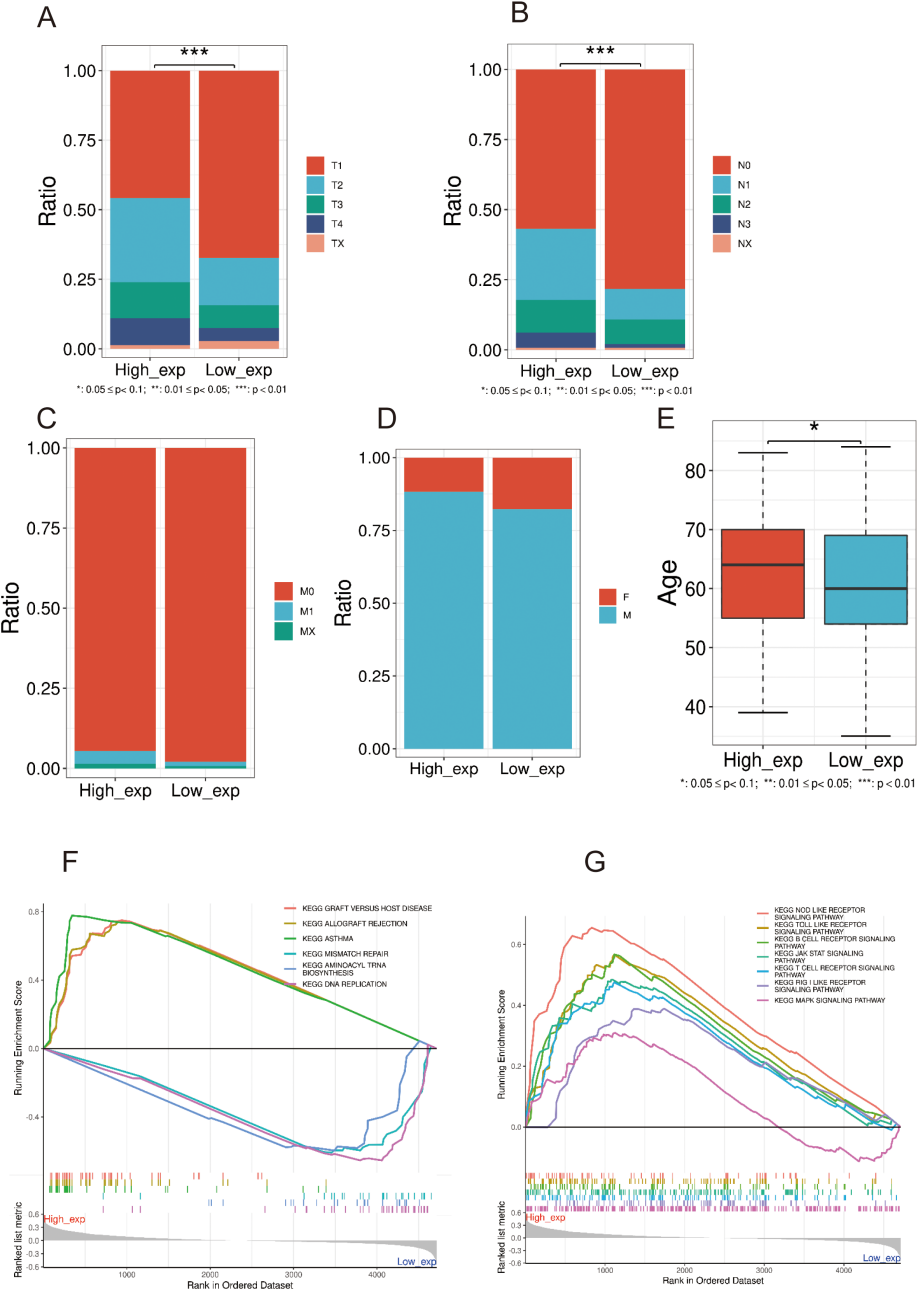
Differences of clinical features in high and low PRMT5 groups. **(A–E)** The expression difference in tumor stage (T, N, and M), sex, and age between the two groups. **(F, G)** GSEA results of DEGs between two PRMT5 groups.

Accordingly, LC samples in GSE30219 data were categorized into two groups (also named high and low PRMT5 group) with the median PRMT5 expression value as the cutoff. We examined the differences in clinical factors between the two PRMT5 subgroups. The results showed that there was a significant difference in tumor stage (T and N) and age while there was no detectable difference in the M stage and gender between the two groups (**Figures 6A–E**). High expression PRMT5 implied an advanced tumor stage. GSEA results showed that the functional pathways of two PRMT5 groups had significant heterogeneity. These pathways including, graft versus host disease, allograft rejection, asthma, mismatch repair, aminoacyl tRNA biosynthesis, and DNA replication were activated in the high PRMT5 group (**Figure 6F**). It was worth noting that immune-related pathways were significantly enriched in the high PRMT5 group, indicating that PRMT5 promoted the activity of immune response (**Figure 6G**).

### 3.7 IC genes related to PRMT5 showed a poor prognosis in LUAD

**Figure 7:**
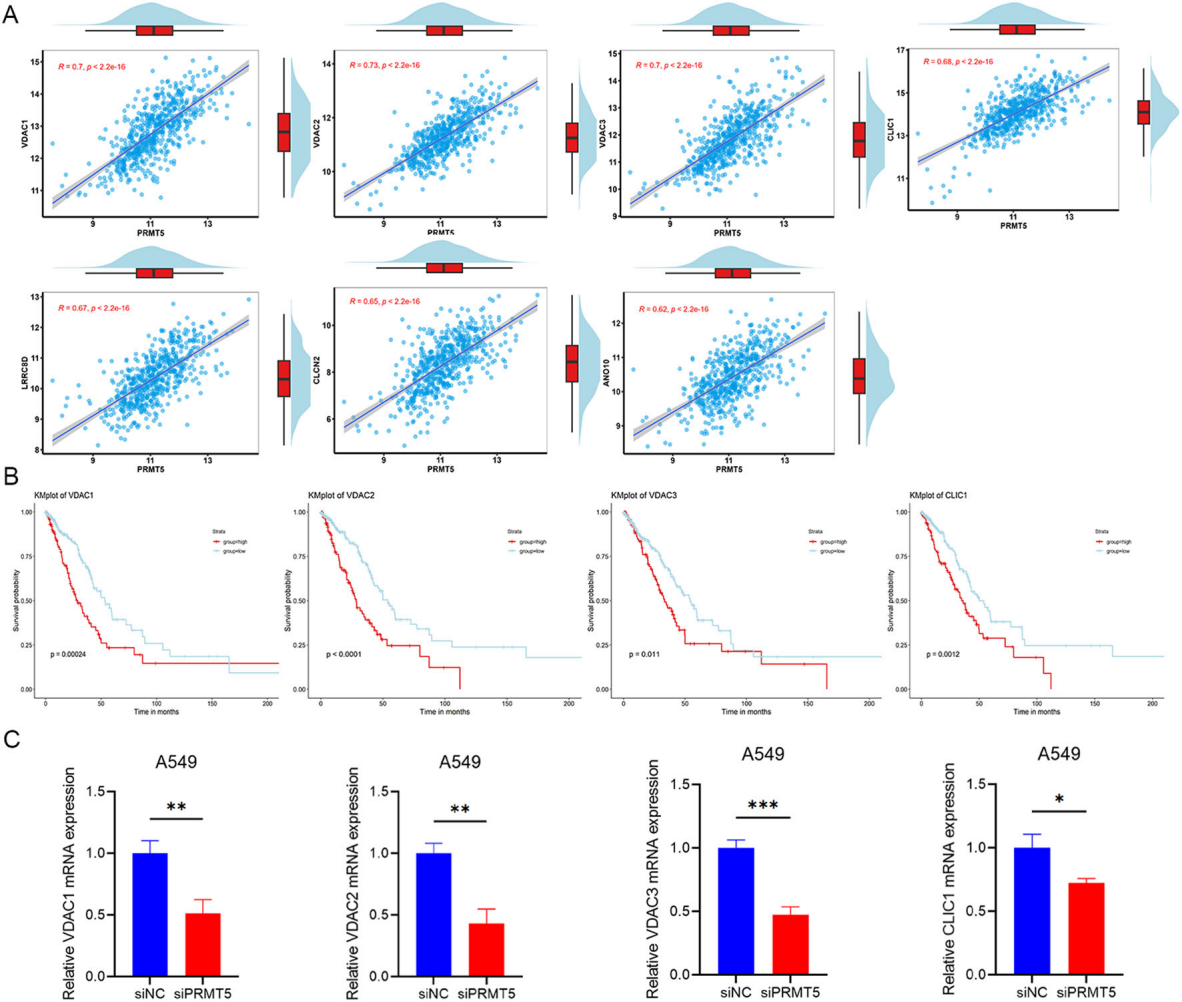
IC genes related to PRMT5 showed a poor prognosis in LUAD. **(A)** The results of pearson correlation between PRMT5 and IC genes (genes withr^2^ |>0.6). **(B)** KM survival analysis results with the four highest hazard ratios of PRMT5-related IC genes. **(C)** Relative mRNA expression of indicated genes was detected in siNC and siPRMT5 groups in A549 cells. (*p < 0.05, **p < 0.01 and ***p < 0.001).

360 IC-related genes were screened and recorded through an extensive literature review, and then compared with 330 genes downloaded from the HUGO Gene Nomenclature Committee (HGNC) database, resulting in a total of 323 different IC-related genes. Subsequently, based on the database of UCSC XEAN (https://xena.ucsc.edu/), pearson correlation was analyzed between IC-related genes and PRMT5 in TCGA-LUAD gene expression data. Secreening genes with | r^2^ |>0.4 as IC genes related to PRMT5 expression, 27 IC related genes were selected including ASIC1, ANO7, ANO8, ANO10, AQP11, CACNB3, CLIC1, CLCN2, CLCN3, CLCN7, CHRNA5, CHRNB1, HCN3, KCND1, P2RX4, TRPM2, TRPM7, MCOLN1, TPCN1, TPCN2, VDAC1, VDAC2, VDAC3, LRRC8A, LRRC8B, LRRC8D and LRRC8E. The results of pearson correlation between PRMT5 and IC genes (genes with | r2 |>0.6) are shown in **Figure 7A**, the other results are shown in **Supplementary Figure S1A**.

To explore the potential interactions between PRMT5-related IC genes, a PPI network was constructed using the STRING database (https://string-db.org/). We have explored the interaction between PRMT5 and these genes, but have not found any correlation.

From the 27 IC genes related to PRMT5 expression, KM survival analysis was performed to detect prognostic significance in LUAD. 9 genes displayed statistical significance and showed a poor prognosis in LUAD, including ASIC1, CLIC1, CLCN3, CHRNA5, KCND1, VDAC1, VDAC2, VDAC3 and LRRC8A. It was deduced that the overexpression of IC genes related to PRMT5 inferred a poor prognosis in LUAD patients. The findings from the KM survival analysis concerning PRMT5-related ICs genes, which exhibited the four highest hazard ratios, were presented in **Figure 7B**. The relative mRNA expression of these four genes was also detected when PRMT5 was knocked down in A549 cells (**Figure 7C**). Additional results can be found in **Supplementary Figure S1B**.

## 4 Discussion

Extensive clinical and preclinical research has indicated that PRMT5 exhibits ectopic expression across various cancer types and is negatively correlated with favorable prognostic outcomes (26). For instance, in glioblastoma (GBM), pharmacological inhibitors of PRMT5 have been shown to reduce the proliferation of GBM stem cells and enhance the sensitivity of proneural subpopulations by altering the splicing patterns of cell-cycle related gene products (27). In CDK4/6 inhibitor-resistant ER+/RB-deficient breast cancer, PRMT5 inhibition represents a viable therapeutic strategy, leading to the dissociation of FUS from RNA polymerase II and subsequently suppressing the DNA synthesis (28). Additionally, PRMT5 promotes the growth of prostate cancer cells by epigenetically binding to the promoter region of the androgen receptor gene (29). Notably, PRMT5 has been conformed to engage in the progression of LC and has a significant impact on the regulation of tumor characteristics. Specifically, PRMT5 has been found to enhance the DNA damage repair response and increase radiosensitivity by facilitating the ubiquitination and degradation of Mxi1 expression in LC (30). Isolated studies have demonstrated that the knockout of PRMT5 in LC cells results in a decreased proliferation rate of cancer cells by disrupting the activity of the Smad7/STAT3 signaling axis (31). These findings remarkedly probe that PRMT5 could serve as a critical regulator in human LC.

In this study, we observed that PRMT5 was upregulated in LC samples compared to normal controls. By establishing PRMT5 overexpression (OE) and wild-type (WT) groups, we determined that PRMT5 is involved in the DNA damage repair response, cell cycle regulation, and the MAPK signaling pathway, corroborating previous findings. These signaling networks have been reported to influence the cell proliferation process by regulating various stages of the cell cycle. A notable example is the activity of the MAPK/ERK/ELK1 signaling pathway, which was distinguished in survival (8), cancer stemness, and the immune microenvironment (32). Furthermore, we identified a strong interactive relationship between PRMT5 and PRDM1. PRDM1, also known as B lymphocyte-induced maturation protein 1 (BLIMP1), has been shown to impair the stemness and growth activity of CAR T cells, as indicated by TCF7, which is closely associated with the exhaustion phenotypes of CAR T cells in anti-tumor therapies (33). Thus, we conclude that PRMT5 is implicated in T cell stemness and functional exhaustion.

Our analysis revealed that elevated levels of PRMT5 expression were negatively correlated with survival outcomes in the LC cohort, as determined through both internal and external datasets. Genomic instability (GI) is an effective prognostic marker of cancer (34). The ROC results indicated that PRMT5 displayed an excellent predictive ability in evaluating the 1-, 3-, and 5-year survival probabilities. The comprehensive prognostic model, which integrated the PRMT5 index alongside other clinical variables such as TNM stage, sex, and age, yielded consistent and insightful results, as detailed in our nomogram analysis. Additionally, existing literature supported the notion that high PRMT5 gene expression was associated with poorer overall survival in LC patients. The small molecule inhibitor AMI-1, which targeted PRMT5, was shown to reduce cell viability and enhance apoptosis by inhibiting the dimethylation of histone 4 (35). Our findings highlighted the crucial function of PRMT5 in LC survival, suggesting its potential as a promising candidate for a biomarker in LC prognosis. Except specific protein biomarkers, exosomal cargo secreted from cancer cells was also associated with the development and progression of LC (36).

To further explore the mechanisms underlying PRMT5’s downstream effects, we categorized LC samples into high and low PRMT5 expression groups. Correlation analyses of clinical characteristics indicated that patients with advanced N and M stages exhibited higher levels of PRMT5 expression, implying that PRMT5 may promote tumor cell metastasis and facilitate cell migration. Emerging evidence has confirmed that PRMT5 enhanced cell proliferation and migration by catalyzing the methylation of the CAMK2N1 promoter sequence, which was regulated by H4R3me2s and H3R8me2s, thereby inhibiting the transcription of CAMK2N1 (26).

ICs have also been demonstrated to play a significant role in the pathogenesis and progression of LUAD (37). While in cancer, the correlation between epigenetic mechanisms and ICs was major studied in pancreatic ductal adenocarcinoma. Notably, DNA methylation in the Ca2^+^ signaling pathway was identified as a factor that facilitated carcinogenesis (32629766). In chronic pain, epigenetic reprogramming underlined cell-specific alternative splicing of calcium ICs, which in turn affected nociceptors (38). Regarding the sense of taste, epigenetic modification led to abnormal gene expression and unique pathophysiological characteristics of ICs (39). Additionally, the disruptor of telomeric silencing could specifically bind to histone H3K79 repressed ENaCalpha transcription (40). Moreover, histone modification could also regulate the expression of K^+^ channels (41, 42).

As for PRMT5, previous research has partially explored the relationship between PRMT5 and ICs. Specifically, PRMT5 has been shown to methylate the cardiac voltage-gated sodium channel, resulting in an increased expression on the cell surface (43). Additionally, PRMT5 could also mediate alternative splicing of transient receptor potential cation channel subfamily M member 4, leading to lower calcium processivity and autoimmune neuroinflammation (44). Melastatin-related transient receptor potential 6 (TRPM6) protein was a protein containing both an IC pore and a serine/threonine kinase. PRMT5’s interaction with the kinase of TRPM6, suggests a potential role in the regulation of TRPM6 expression (45). Furthermore, P2X5, a member of ligand-gated IC receptors, was associated with osteoclast maturation. Methylosome protein 50, a crucial cofactor of PRMT5, was associated with P2X5 to regulate the osteoclast maturation, indicating a link between the PRMT5 complex and P2X5 signaling pathway (46). Moreover, PRMT5 was also reported to increase the expression of transient receptor potential cation channel, subfamily V, member 6 and then subsequently influence cancer cell proliferation, apoptosis and autophagy (47). However, the relationship between PRMT5 and ICs is still unclear and warrants further systematic studies.

Furthermore, based on the literature and HGNC starbase, we found 323 genes related to ICs, and then combined with the data of LUAD, 27 ICs-related genes were selected including ASIC1, ANO7, ANO8, ANO10, AQP11, CACNB3, CLIC1, CLCN2, CLCN3, CLCN7, CHRNA5, CHRNB1, HCN3, KCND1, P2RX4, TRPM2, TRPM7, MCOLN1, TPCN1, TPCN2, VDAC1, VDAC2, VDAC3, LRRC8A, LRRC8B, LRRC8D and LRRC8E. Our results expand the scope of IC genes related to PRMT5 and provide new insight into the role of PRMT5. 9 genes were identified as statistically significant and correlated with poor prognosis in LUAD, including ASIC1, CLIC1, CLCN3, CHRNA5, KCND1, VDAC1, VDAC2, VDAC3 and LRRC8A. IC genes related exhibit a variety of functions. Anoctamin (ANO) protein family (ANO7, ANO8, and ANO10) was the molecular basis of the Calcium-activated chloride channel. CLIC1, a Chloride intracellular channel, was overexpressed in LUAD and was associated with tumor metastasis, tumor staging, and OS (48). CLCN3 was one of the members of the Chloride channel family, upregulated in LUAD, and CLCN3 knockdown could inhibit LUAD proliferation and migration (49). CHRNA5, which encoded the alpha5 nicotinic acetylcholine receptor, was not only associated with the progression of LUAD (50, 51), but also mediating EGFR signaling pathway to regulate the sensitivity to gefitinib *in vitro* and vivo (52). VDAC1, VDAC2 and VDAC3 were all significantly associated with LUAD prognosis (53, 54). In addition, the knockdown of VDAC3 significantly increased the sensitivity to cisplatin in LUAD cells and had assistance in neoadjuvant chemotherapy (53). Additionally, PRMT5 was also reported to regulate cisplatin chemosensitivity (35), suggesting that one potential mechanism by which PRMT5 influenced cisplatin response may involve VDAC3.

Our team noticed that LRRC8A was an essential component of the volume-regulated ion channel, associated with poor prognoses in colon cancer patients by enhancing cancer cell proliferation and metastasis (55–57). The initial acquisition rather than the maintenance of oxaliplatin resistance was also promoted by LRRC8A (58). Our research predicted LRRC8A was also a poor prognostic biomarker in LUAD, expanding the scope of LRRC8A’s function in prognosis prediction. The relationship between PRMT5 and LRRC8 family has not been studied, our research provided new evidence to clarify the relationship between PRMT5 and LRRC8A, LRRC8B, LRRC8D as well as LRRC8E.

Our research provides evidence that except anti-tumor effect, PRMT5 inhibitor may also be applied to the treatment of various diseases related to ICs, such as chronic pain, dysgeusia, disorders affecting nervous system, muscles, heart, kidneys and so on. However, the primary application of PRMT5 inhibitors remains focused on anti-tumor therapies at this time. Consequently, it is crucial to recognize that the abnormal phenomenon related to ICs may also manifest as side effects of PRMT5 inhibitors, which may pose significant challenges to the clinical translation of these agents.

## 5 Conclusion

Collectively, our findings underscored the functional effect of PRMT5 and provided a rationale for targeting PRMT5 in LC treatment. PRMT5 emerges as a potential biomarker for prognostic assessment in LC. Furthermore, the interplay between PRMT5 and PRDM1 may be instrumental in the advancement of LC. Additionally, PRMT5 was also involved in the regulation of IC genes, associated with membrane potential and progression of LUAD cells.

## Data Availability

The datasets presented in this study can be found in online repositories. The names of the repository/repositories and accession number(s) can be found in the article/**Supplementary Material**.
