# Supplementary material for: A comprehensive investigation of PRMT5 in the prognosis and ion channel features of lung cancer
Source: Front Oncol. 2024 Nov 29;14:1478672. doi: 10.3389/fonc.2024.1478672 (PMC11638061; doi:10.3389/fonc.2024.1478672)
Supplement: Supplementary file 1 [file DataSheet1.docx]

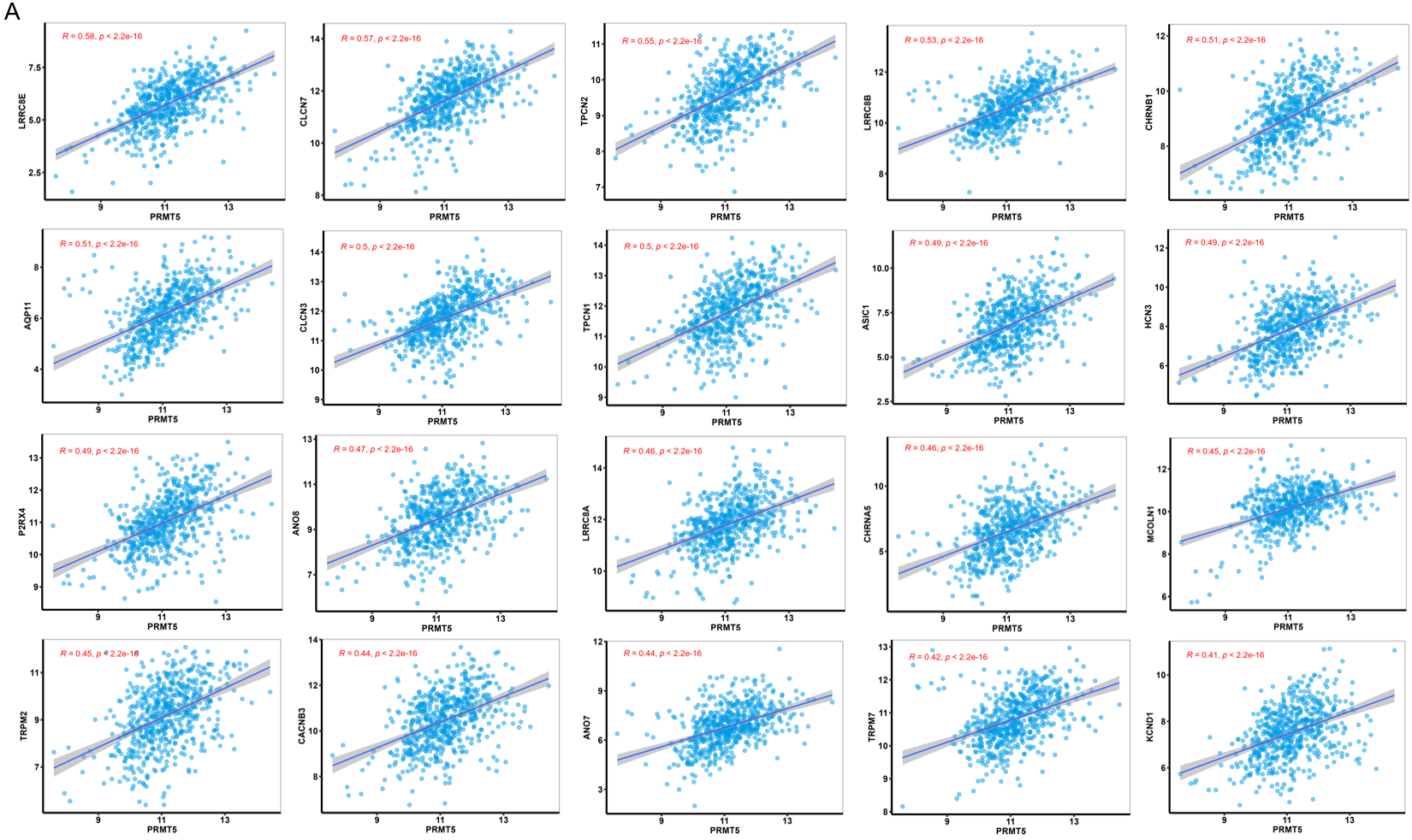

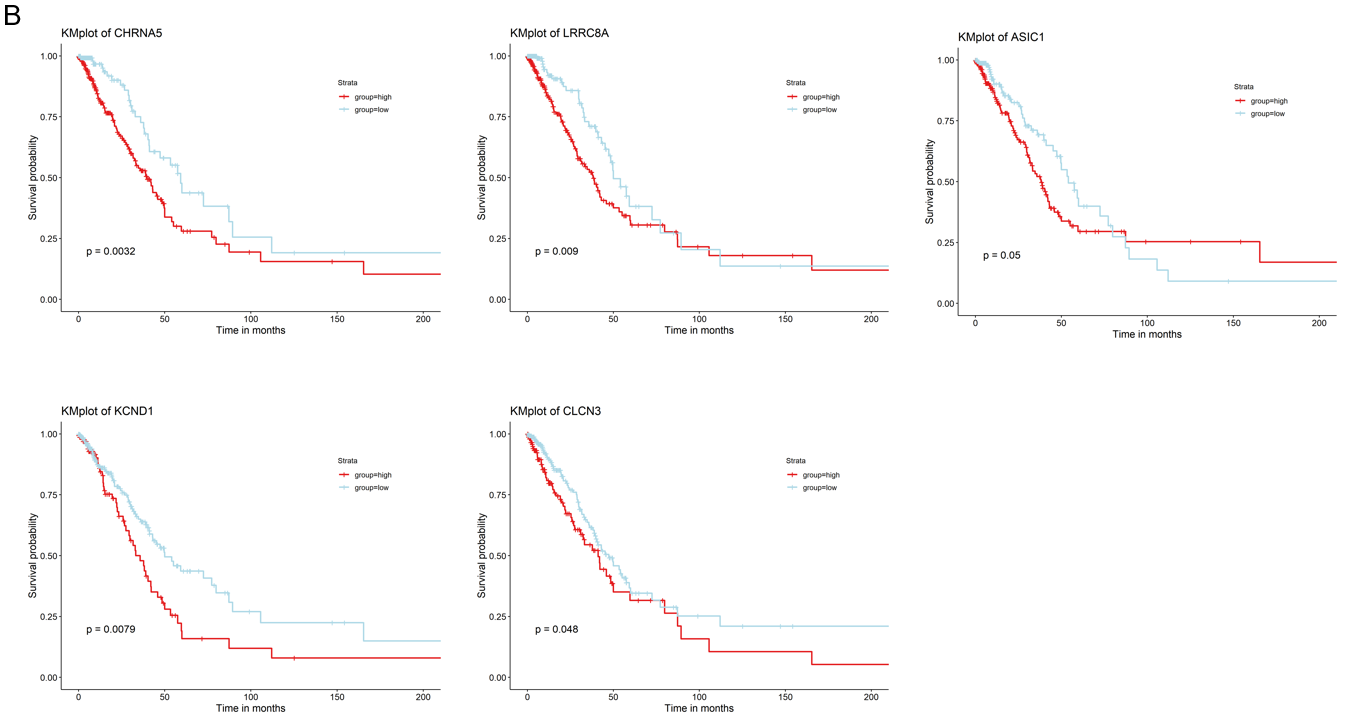


Fig S1. (A) The results of pearson correlation between PRMT5 and ion channel genes (genes with 0.4<| r^2^ |<0.6). (B) The other part of the results of KM survival analysis of PRMT5-related ion channel genes.
